# Flavour stability of sterilised chickpeas stored in pouches

**DOI:** 10.1016/j.crfs.2021.10.011

**Published:** 2021-10-30

**Authors:** Laura E.C. Noordraven, Mikael A. Petersen, Ann M. Van Loey, Wender L.P. Bredie

**Affiliations:** a KU Leuven, Laboratory of Food Technology (member of Leuven Food Science and Nutrition Research Centre, LFoRCe), Department of Microbial and Molecular Systems (M^2^S), Kasteelpark Arenberg 23 Box 2457, 3001, Leuven, Belgium; b University of Copenhagen, Design and Consumer Behaviour, Department of Food Science, Faculty of Science, University of Copenhagen, Rolighedsvej 26, 1958, Frederiksberg C, Denmark

**Keywords:** *Cicer arietinum* L., GC-MS-O, Sensory descriptive analysis, Shelf-life, Sterilised chickpeas, Packaging materials

## Abstract

The increasing need for sustainable food choices places a demand on developing palatable foods from lower impact production and with a suitable shelf-life. In this context, knowledge of the sensory properties of whole sterilised chickpeas is required to be able to make them more attractive to the consumers. The sensory quality of chickpeas is largely dependent on the aroma and flavour, which can be influenced by storage conditions. In this study, sterilised chickpeas in two different packaging materials with different oxygen permeabilities, stored up to 52 weeks at ambient temperature (20 °C) were investigated using both descriptive sensory profiling and gas chromatography – mass spectrometry – olfactometry analysis (GC-MS-O). During storage, intense, sulphury and meat-like aromas decreased in intensity, while with longer storage time hay-like, green-like and potato-like flavours and aromas became more apparent. A total of 40 odour-active volatiles were detected, of which some had properties reminiscent of the chickpea flavour profiles. However, no clear relationships could be established between these odorants and the sensory changes observed during storage by descriptive sensory analysis. It was concluded that, significant changes in the sensory attributes of sterilised chickpeas occurred during 52 weeks of storage, but that packaging material does not seem to largely influence the sensory attributes during storage.

## Highlights

• Significant sensory changes occur in sterilised chickpeas during 52 weeks storage.
• 40 odour-active volatiles are detected in the sterilised chickpeas.
• Sulphur compounds formed during sterilisation change during storage.
• Hay and green attributes are perceived more intense with increased storage time.
• Oxygen permeability of packaging does not impact the sensory quality during storage.

## Graphical abstract

**Figure:**
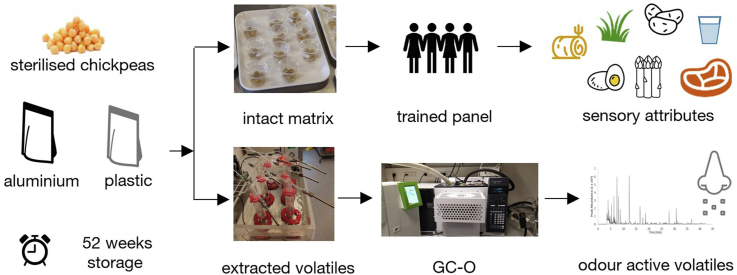

## 1 Introduction

Legumes, such as chickpeas, common beans and lentils, are an important part of the diet of low-income groups in developing countries (Liu et al., 2006). In general, legumes have a high protein content, contain vitamins and micronutrients (e.g. iron and zinc) in considerable concentrations and are a good source of complex carbohydrates (e.g. starch) (Wang et al., 2010). Furthermore, they are considered as a relatively cheap and more sustainable source of proteins compared to animal products. Therefore, chickpeas and other legumes are becoming increasingly popular.

Chickpeas are often sterilised, resulting in ready-to-use chickpeas with a relatively long shelf-life. During the shelf-life period, food should remain safe, and keep its desired sensorial, chemical, physical and functional characteristics (Fu and Labuza, 1997). Sensory changes in sterilised foods during storage depend on several factors, such as oxygen availability, light exposure, storage temperature and storage time. Generally, sterilised chickpeas are stored at room temperature, protected from direct light.

Oxygen availability during storage influences the non-enzymatic lipid oxidation reactions taking place during storage of legumes such as sterilised chickpeas, peanuts and roasted marama beans (Holse et al., 2012; Jensen et al., 2005; Noordraven et al., 2021). The volatile profile of sterilised chickpeas during storage at different oxygen availabilities has been previously studied. Although the volatile changes at room temperature appeared to be low, some different reactions were observed during storage in different packaging materials with different oxygen permeabilities. At higher oxygen levels more hydrocarbons, sulphur compounds and ketones were formed, whereas at lower oxygen levels more alcohols were observed (Noordraven et al., 2021). Volatile compounds formed during oxidation reactions can give rise to new volatiles, which can influence sensory acceptability of the food (Jensen et al., 2005). Factors such as flavour and aroma largely determine the overall acceptability of sterilised chickpeas. Unfortunately, the aroma and flavour of legumes are not always appreciated by consumers (Xu et al., 2019). Unfortunately, hitherto no studies on the aroma active compounds in chickpeas, or on the flavour changes in sterilised chickpeas during storage have been reported. Since food sustainability is getting increasingly important, a more precise understanding of the sensory properties of sterilised chickpeas is needed, facilitating the possibility to make them more attractive to consumers.

A commonly used technique to assess sensory differences in food products is descriptive sensory analysis (Murray et al., 2001). This technique has been used for sensory evaluation of several chickpea-related products, such as chickpea flour enriched biscuits and gels, steamed and toasted chickpea flour and puffed chickpea snacks (Jiménez et al., 2016; Mukhopadhyay et al., 2018; Rababah et al., 2006; Ravi et al., 2011). Despite the need for an increased legume consumption and the growing consumer interest in chickpeas, little is known about the sensory profile of sterilised chickpeas. Furthermore, the flavour compounds contributing to the sensory profile of chickpeas have not been reported.

Using gas chromatography–mass spectrometry–olfactometry (GC-MS-O) analysis, odour-active volatile compounds that elute from the GC-column can be assessed by a human panel via a sniffing port. The advantages of GC-MS-O analysis are that key impact volatiles can be determined and that the human nose can detect compounds with very low odour thresholds, which might not be recognised using solely GC-MS. Disadvantages are that not all humans are able to smell all volatile components, hence a panel of multiple trained judges is needed (Reineccius, 2010). Additionally, many volatiles show concentration dependent odour characteristics, meaning that at different concentrations, specific volatiles may be perceived as having different odours (Bott and Chambers, 2006). Since volatiles might be concentrated during GC-MS-O analysis and because only part of the molecules go to the sniffing port, it is possible that volatiles are differently observed in the GC-MS-O compared to the real food (Reineccius, 2010). Additionally, combinations of volatiles and interactions with other food compounds in the real foods might give a different sensory experience (Bott and Chambers, 2006).

Several different GC-MS-O approaches have been described, including detection frequency, perceived intensity, odour profiling and detection thresholds methods (Petersen et al., 2003). These GC-MS-O methods create the opportunity to correlate the sensory description of foods to its volatile profile. Hitherto, the odour-active compounds in sterilised chickpeas have not been described in literature.

Therefore, present study investigated the sensory properties of whole sterilised chickpeas during storage by means of descriptive sensory profiling and GC-MS-O. Both the effects of storage time and oxygen availability were considered, using two different packaging materials (plastic pouches and aluminium coated pouches) and storage times up to 1 year at room temperature (20 °C).

## 2 Materials and methods

### 2.1 Materials

The kabuli chickpeas (*Cicer arietinum* L.) used were supplied by Greenyard Prepared (Bree, Belgium). The chickpeas originated from Argentina, harvested late December 2018. The dried chickpeas were stored at -40 °C until the day of use.

Two different packaging materials were used in this study (Amcor Flexibles, Moreuil, France). These two packaging types, further referred to as ‘plastic pouches’ and ‘aluminium pouches’ were composed of three layers (from outside to inside: 12 μm polyester, 12 μm polyethylene terephthalate with silicon oxide and 70 μm polypropylene) and four layers (from outside to inside: 12 μm polyester, 9 μm aluminium, 15 μm oriented polyamide and 70 μm polypropylene), respectively. The oxygen permeability at 23 °C was 1 and 0.05 cm³/(m^2^day.bar), for the plastic and aluminium pouches, respectively.

All food grade taste standards (caffeine, sucrose and L-glutamic acid monosodium salt monohydrate) and all chemical analytical standards were obtained from Sigma-Aldrich (St. Louis, Missouri, United States).

### 2.2 Production and storage of the sterilised chickpeas

Sterilised chickpeas were obtained as described in Noordraven et al. (2021). Frozen dried chickpeas were soaked in an excess of standardised production water of Greenyard Prepared (Bree, Belgium) (drinking water quality) at room temperature for 16 h. Afterwards, the soaked chickpeas were filled into the plastic and aluminium pouches together with the standardised production water of Greenyard Prepared (ratio soaked chickpea:water 2:3) and pouches were sealed with minimal headspace. Pouches were subsequently sterilised for 40 min at 116 °C using a Steriflow pilot retort (Barriquand, Paris, France). The coldest spot of the pouches reached an F_0_-value of 15.6 min, to obtain a palatable texture.

The aluminium and plastic pouches with sterilised chickpeas were stored in the dark in temperature-controlled incubators at 20 °C. Samples were collected at 7 different time points (0, 1, 4, 8, 16, 32 and 52 weeks).

For the samples used in the descriptive sensory test, the pouches were opened in a food grade environment, the aquafaba was drained, the chickpeas were transferred into food grade freezer bags and stored at -20 °C until the day of analysis. Samples from both packaging materials and all 7 time points were included in the descriptive sensory test (14 samples in total). For the chickpeas used for the GC-MS-O analysis, pouches were placed in an ice bath for 30 min prior to opening. Subsequently, they were opened in a temperature-controlled cold room (4 °C), aquafaba was drained and chickpeas were placed in odourless tubes, frozen with liquid nitrogen and stored at -20 °C. For the GC-MS-O tests, the extreme time points (0 and 52 weeks) of both packaging materials were included (4 samples in total). All samples were kept in the freezer at -20 °C from their respective sampling times and were analysed after the final storage time was reached.

### 2.3 Sensory descriptive analysis

#### 2.3.1 Sample preparation

Prior to the sensory analysis, five frozen chickpeas were transferred into transparent, odourless food grade 30 ml cups (polypropylene) with lid (amorph polyethylene terephthalate) (Sæbe Compagniet ApS, Herlev, Denmark). Chickpeas were thawed for at least 1 h in a temperature-controlled incubator at 20 °C. All samples were served at 20 °C, labelled with a randomised three-digit code.

#### 2.3.2 Sensory evaluations

A descriptive sensory profiling was used to determine the influence of storage time and oxygen availability on the aroma and flavour of sterilised chickpeas. Ten assessors (8 female/2 male, aged between 22 and 47 years) were recruited from the sensory panel at the University of Copenhagen (UCPH). The assessors were screened for sensory perception sensitivity prior to the panel selection and had previous experience with participating in descriptive sensory studies. The sensory profiling was performed in the sensory laboratory of UCPH under standardised conditions and in accordance with ISO standard 67.240 for sensory analysis. All assessors were asked to sign an informed consent form prior to participating in the test.

The sensory profiling was performed over 6 days (4 training days and 2 evaluation days) over a time span of 10 days. During the first training session potential sensory attributes were developed by the panel. References were used to reach consensus among assessors on the meaning of the sensory attributes (Table 1). The last training days were focused on the consensus in intensity ranking of the different attributes.

**Table 1:** Sensory attributes used in the descriptive analysis, including scale anchors and reference materials.

Modality	Attribute	Scale Anchors^b^	Reference
Aroma^a^ (A) (1st sniff)	Overall Intensity	None-a lot	–
	Sulphury	None-a lot	Boiled eggs
Aroma^a^ (A) (2nd sniff)	Green	None-a lot	Raw sugar snap
	White Asparagus	None-a lot	Brine from jarred asparagus and water (ratio 1:5)
	Boiled Potato	None-a lot	Boiled potato
	Hay	None-a lot	Rye kernels
	Meat Broth	None-a lot	Boiled beef and cooking water
Basic Taste (BT)	Sweet	Little-a lot	Sucrose solution (7.2 g/L)
	Bitter	Little-a lot	Caffeine solution (0.549 g/L)
	Umami	None-a lot	L-glutamic acid monosodium salt monohydrate solution (0.7 g/L)
Flavour (F)	Watery	Little- a lot	Boiled bulgur
	Corn	None-a lot	Corn brine from can and water (ratio 1:4)
	White Asparagus	None-a lot	Brine from jarred asparagus and water (ratio 1:5)
	Hay	None-a lot	Rye kernels
	Oxidised	None-a lot	Rustic potato chips (bag opened overnight)
	Boiled Potato	None-a lot	Boiled potato
	Meat Broth	None-a lot	Boiled beef and cooking water

a orthonasal aroma, assessed by sniffing the sample.

b “a lot” is translated from the Danish word “meget”, which is the common way of expressing intensity in the Danish language.

The chickpea samples were evaluated using a 15 cm unstructured line scale using FIZZ software (version 2.15 c02, Biosystemes, Courtenon, France).

Assessors received a closed plastic container with five sterilised chickpeas, which they were instructed to rotate end-over-end for 3 times and afterwards to open the container and to take one deep sniff. The cups were immediately closed and the panel judged upon the aroma attributes for ‘first sniff’. This procedure was repeated for the second sniff. Afterwards, the assessors were instructed to taste the chickpeas, where at least 2 of the 5 chickpeas had to be tasted (to counteract one-to-one variation). Now, the assessors judged upon the taste and flavour attributes.

Once assessors received the next sample, they were not able to evaluate the previous sample again or change their previous answers. Assessors were instructed to rinse their mouth with water between each sample.

The evaluation was carried out in individual sensory booths using a randomised block design for the serving order. Each assessor evaluated each sample four times in four different evaluation sessions.

#### 2.3.3 Data analysis

Panel performance during training sessions was monitored using PanelCheck (version 1.4.2, Nofima, Norway), using the profile plots and Tucker-1 plots functions. Samples were checked for outliers, by visual inspection of the plots of individual sensory attributes, and by plotting the data per attribute and compare estimated model data to actual values.

The data were analysed as three data sets: i) samples from the plastic pouches, ii) samples from the aluminium pouches, and iii) all samples of both packaging materials together. A general linear mixed Analysis of Variance (ANOVA) model was performed, with sample effect as fixed factor and the assessor and replicate effects as random factors. For significant fixed effects, the ANOVA was followed by Tukey's Honestly Significant Difference (HSD) post hoc test (P<0.05). The analysis was performed using Statistical Package for the Social Sciences (SPSS 25.0, IBM, Armonk, NY, United States).

The software SOLO (Version 8.7.1, 2020, Eigenvector Research, Inc., Manson, WA, United States) was used to perform principal component analysis (PCA) on the mean score values (per repetition) of the attributes which were found to be significantly changing over storage time. All data were autoscaled prior to obtaining the PCA model. Selection of the number of principal components (PCs) in the PCA models was based on the explained variance and the root mean square error of cross validation (RMSECV). Biplots of two PCs were generated using the software OriginPro (OriginPro 8, OriginLab Corporation, Northampton, MA, United States) to visually represent the established PCA models.

### 2.4 Gas chromatography- mass spectrometry - olfactometry analysis

#### 2.4.1 Dynamic headspace extraction

Dynamic headspace extraction was carried out using a method adapted from Qin et al. (2018)*.* On the day of extraction, chickpea samples were defrosted for 20 min in a water bath at 20 °C and subsequently mixed with demineralised water (ratio 1:1). From this point onwards the sample was always kept in an ice bath until the extraction, to avoid volatile losses. Using an Ultraturrax (T25 digital, Janke & Kunkel, IKA Labortechnik, Staufen, Germany) at 12000 rpm for 1 min, the mixture was homogenized into a chickpea puree.

Of this puree, 7.5 g, together with 0.2 ml of internal standard solution (4-methyl-1-pentanol, 5 mg/L in destilled water) were added to gas washing flasks (150 ml) and equilibrated in a 40 °C circulating water bath under magnetic stirring at 200 rpm. The samples were purged with nitrogen for 40 min (purge flow: 200 ml/min). Volatiles were trapped on Tenax TA traps with mesh size 60/80 (±250 mg, Markes International, Llantrisant, UK), which had been cleaned using a tube conditioner (TC-20, Markes international Ltd, UK) for 55 min at 300 °C. Water was removed from the traps by dry nitrogen purge of 20 min (purge flow: 200 ml/min). Prepared traps were stored at 4 °C until the GC-MS-O analysis.

#### 2.4.2 Gas chromatography – mass spectrometry – olfactometry procedure

The desorption and GC heating profiles were adapted from Qin et al. (2018)*.* Volatiles were desorbed from the Tenax traps using a thermal desorber (TurboMatrix 300, PerkinElmer Inc, USA). Primary desorption took place at 250 °C for 20 min with H_2_ as carrier gas at a flow of 50 ml/min. Volatiles were collected on a cold trap (Tenax TA with mesh size 60/80, ±50 mg, Markes International, Llantrisant, UK) that was held at 5 °C. During the secondary desorption, the cold trap was heated to 300 °C at 99 °C/min and held for 2 min.

Volatiles were transferred in splitless mode via a heated transfer line (225 °C) to a GC-MS system (7890B, Agilent Technologies, USA) coupled to a MSD 5977 (Agilent Technologies, USA) and an olfactory detection port (ODP2, Gerstel GmbH & Co., Germany) at an inlet pressure of 179 kPa at a flow rate of 22.6 ml/min.

A DB-WAX column (Agilent Technologies, USA) (30 m, 0.25 mm, 0.25 μm, LTM) was used to separate the volatiles, with H_2_ as carrier gas, at a constant flow of 2.1 ml/min and an inlet pressure of 179 kPa and an outlet pressure of 134 kPa. The temperature profile of the GC oven started with a holding step at 40 °C for 10 min, followed by a temperature ramp of 8 °C/min until 240 °C was reached, and finally a holding step at 240 °C for 5 min. At the end of the GC column, the outlet was split to the MS and the sniffing port in a ratio (1.7:5.8). MS detection was obtained by electron ionisation mode at 70 eV with a scanning range of 15–300 m/z. The MS ion source and quadrupole were set at 230 °C and 150 °C, respectively. The olfactory detection port was supplied with humidified air.

#### 2.4.3 Odour evaluation

A panel of 9 judges (2 male/7 female, aged between 24 and 54 years) was used in the GC-MS-O study. Panellists were trained during two training sessions, to get used to the sniffing procedure and the use of the scale. In these training sessions, panellists were asked to sniff a test mixture, consisting of known compounds (aromas) and intensities. During the evaluation sessions, each chickpea sample was assessed once by each panellist. Panellist were asked to indicate when they perceived an odour, and the character and overall odour intensity of every perceived odour on a 6-point scale adapted from the labelled magnitude scale, in which all intensity scores were at equal distance (0: not detected, 1: very weak odour (just noticeable), 2: weak odour; 3: moderate odour (clearly recognisable but not intense), 4: strong odour (intense), 5: very strong odour (very intense)) (Green et al., 1993). Panellist indicated clearly when a new odour started, to insure discrete values for odour-active compounds.

Perceived odour intensities were block-centred to counteract individual scaling differences between assessors using eq. (1).

[Eq. 1] $$I_{X\_blockcentered}=I_{X}-\left(I_{X\_average}-I_{average}\right)$$

I_X_blockcentred_ is the block-centred intensity score for a compound given by assessor X, I_X_ is the original intensity score for a compound given by assessor X, I_X_average_ is the average intensity score of all compounds scored by assessor X and I_average_ is the average intensity score of all compounds scored by all assessors.

Volatile compounds were considered odour-active when more than three panellists could detect the compound in at least one sample. The Nasal Impact Frequency (NIF) per compound was determined per odour-active compound using Eq. (2), as described by Pollien et al. (1997).

[Eq. 2] $$NIF=\frac{P_{detected}}{P_{total}}\times 100\%$$

P_detected_ is the number of panellists that have detected the compound and P_total_ the total number of panellists (9). The intensity score (IS) per compound was determined using Eq. (3).

[Eq. 3] $$IS=\frac{I_{blockcentered\_average}}{I_{max}}\times 100\%$$

I_blockcentred_average_ is the average of the block-centred intensities of a compound scored by all assessors and I_max_ is the maximum intensity (5). In order to describe the odour importance (OI (%)) of the odour-active volatiles, the geometric mean of the NIF and IS was taken (Eq. (4)) (Wei et al., 2020).

[Eq. 4] $$OI=\sqrt{NIF\times IS}$$

#### 2.4.4 Volatile compound identification

The obtained chromatograms were analysed using the PARAFAC2-based software PARADISe (v3.88, University of Copenhagen, Copenhagen, Denmark) (Johnsen et al., 2017) for peak deconvolution and peak identification based on the comparison of the mass spectra to reference mass spectra in the spectral library of NIST (NIST11, version 2.0, National Institute of Standards and Technology, Gaithersburg, MA, USA). Retention Indices (RI) were calculated based on the retention times of alkane standards (C_6_–C_22_) that underwent the same GC-MS-O procedure as described in 2.4.2. To confirm compound identifications, the RI and odour descriptions of the compounds were compared to values reported in literature. Moreover, when available, comparison of mass spectrum, RI and odour with an analytical standard was performed. When an odour was perceived by the panel, but no peak was visible in the chromatograms, the compound was tentatively identified based on aroma descriptions and RI values of analytical standards and/or values reported in literature. In present study, potential co-eluting compounds were not checked on a second (non-polar) column.

## 3 Results and discussion

### 3.1 Sensory descriptive analysis

#### 3.1.1 Attributes for sensory analysis

A list of attributes was established by the panel, including aroma (divided in first and second sniff), basic taste and flavour attributes. The final attribute list and the respective references used during the sensory study are given in Table 1. These attributes give an overall description of sterilised chickpea samples. In addition to this final attribute list, other attributes were mentioned by the panel. These attributes included spicy, dusty, roasted, popcorn, nutty and earthy aromas and a slight metallic and bitter after taste. Although these attributes contributed to the sensory description of chickpeas, they were not included in the final list as they were considered either to be correlating with other attributes or to remain constant during storage.

#### 3.1.2 Sample and panel outliers

The chickpeas stored for 8 weeks in the aluminium packaging was determined as outlier and excluded from the data (data not shown). No particular panellist was excluded based on the Tucker-1 analysis of the sensory profiling (data not shown). However, the panellists were an important source of variation in several attributes. For some attributes, there were significant replicate effects and/or assessor*sample interactions. This was considered during the determination of attribute effects.

#### 3.1.3 Sensory profiling of chickpeas stored up to 52 weeks

##### 3.1.3.1 Influence of storage time on the sensory attributes of sterilised chickpeas

Six different sterilised chickpea samples stored in aluminium pouches, and seven in plastic pouches, with storage times between 0 and 52 weeks, were assessed by the panel. Of the 17 attributes evaluated, 14 attributes changed during storage in at least one packaging material. This indicated that over the 52-week storage period significant sensory changes were induced in sterilised chickpeas.

For most attributes, in at least one packaging material significant changes were found during storage, while only bitter taste and corn and boiled potato flavour remained constant in both packaging materials (Table 2 and Table 3, for aluminium and plastic pouches respectively). Additionally, in the aluminium pouches, boiled potato aroma, sweet taste, watery flavour and white asparagus flavour remained constant, whereas in the plastic pouches this was the case for oxidised flavour and white asparagus aroma.

**Table 2:** Mean scores* and standard deviations of sensory attributes for chickpeas stored up to 52 weeks in aluminium pouches. A = orthonasal aroma, BT = basic taste, F = flavour. Standard deviations are based on 4 repetitions. Values indicated by a different letter are significantly different (α = 0.05). W = weeks of storage.

Sensory Attributes	Storage time					
	0 W	1 W	4 W	16 W	32 W	52 W
A_Overall Intensity	9.1 ± 0.9 ab	10.6 ± 0.5a	8.5 ± 0.9b	7.9 ± 0.9b	7.4 ± 0.5b	8.2 ± 1.1b
A_Sulphury	6.1 ± 1.3 ab	8.5 ± 0.9a	5.0 ± 1.2bc	4.6 ± 1.6bc	2.7 ± 0.4c	3.1 ± 0.6c
A_Green	3.4 ± 0.4 ab	3.3 ± 0.3b	3.8 ± 0.2 ab	3.3 ± 0.8 b	4.3 ± 1.0 ab	5.0 ± 0.8a
A_White Asparagus	6.4 ± 1.1a	6.1 ± 0.8 ab	5.4 ± 0.7 ab	4.9 ± 0.7 ab	5.7 ± 0.8 ab	3.8 ± 0.3b
A_Boiled Potato	5.5 ± 0.2a	5.0 ± 0.8a	5.5 ± 1.5a	4.6 ± 0.5a	5.7 ± 0.6a	5.0 ± 1.0a
A_Hay	5.2 ± 0.2c	6.1 ± 0.6abc	5.6 ± 0.9bc	5.6 ± 1.1bc	8.6 ± 1.5 ab	9.2 ± 0.8a
A_Meat Broth	8.6 ± 1.1a	9.2 ± 0.6a	7.1 ± 1.4 ab	6.9 ± 0.4 ab	4.6 ± 0.9bc	3.7 ± 0.4c
BT_Sweet	7.3 ± 1.0 a	5.8 ± 0.9a	6.4 ± 0.6a	5.5 ± 1.0a	6.1 ± 0.6a	6.1 ± 1.3a
BT_Bitter	5.6 ± 0.6a	5.2 ± 0.3a	5.4 ± 1.1a	5.4 ± 1.6a	5.6 ± 0.9a	6.6 ± 0.8a
BT_Umami	8.4 ± 0.5a	8.1 ± 0.5a	8.1 ± 0.7a	6.7 ± 1.1 ab	6.7 ± 1.5 ab	5.5 ± 0.7b
F_Watery	5.2 ± 0.5a	5.1 ± 1.0a	6.5 ± 0.8a	7.0 ± 0.5a	6.9 ± 0.5a	5.0 ± 0.7a
F_Corn	5.9 ± 0.5a	5.3 ± 0.8a	5.2 ± 1.0a	4.4 ± 1.0a	5.7 ± 0.6a	4.8 ± 0.7a
F_White Asparagus	5.3 ± 0.5a	6.3 ± 0.8a	5.4 ± 0.3a	4.7 ± 0.6a	5.5 ± 0.6a	4.5 ± 1.3a
F_Hay	6.8 ± 0.5 ab	6.9 ± 1.0 ab	6.7 ± 1.1 ab	6.6 ± 0.8b	8.6 ± 0.7 a	8.3 ± 1.1 ab
F_Oxidised	2.8 ± 0.3b	2.5 ± 0.8 b	2.2 ± 1.2b	2.1 ± 0.5b	2.9 ± 0.7 b	7.1 ± 1.0a
F_Boiled Potato	8.4 ± 0.7a	8.3 ± 0.6a	8.7 ± 0.5a	8.6 ± 1.5a	9.1 ± 0.5a	7.6 ± 0.6a
F_Meat Broth	6.5 ± 0.6 ab	6.9 ± 0.7a	5.8 ± 1.3 ab	4.8 ± 0.8 ab	5.0 ± 1.3 ab	3.9 ± 0.2b

*Panel averaged scores on the 15 cm intensity scale.

**Table 3:** Mean scores* and standard deviations of sensory attributes for chickpeas stored up to 52 weeks in plastic pouches. A = orthonasal aroma, BT = basic taste, F = flavour. Standard deviations are based on 4 repetitions. Values indicated by a different letter are significantly different (α = 0.05). W = weeks of storage.

Sensory Attributes	Storage time						
	0 W	1 W	4 W	8 W	16 W	32 W	52 W
A_Overall Intensity	13.1 ± 0.3a	12.1 ± 0.5a	7.8 ± 0.8b	7.2 ± 1.0b	6.4 ± 0.7b	7.0 ± 0.9b	6.8 ± 0.2b
A_Sulphury	12.7 ± 0.4a	11.7 ± 0.5a	5.5 ± 1.5b	3.3 ± 0.8bc	1.7 ± 1.7c	2.1 ± 0.9c	1.7 ± 0.6c
A_Green	3.0 ± 0.3c	2.8 ± 0.4c	3.8 ± 0.3bc	4.8 ± 0.4 ab	4.8 ± 1.3 ab	6.1 ± 0.8a	5.7 ± 0.3a
A_White Asparagus	6.2 ± 0.6a	6.0 ± 1.2a	5.0 ± 1.0a	5.0 ± 1.3a	4.5 ± 0.6a	4.6 ± 0.5a	4.7 ± 0.5a
A_Boiled Potato	4.1 ± 0.5b	5.1 ± 0.4 ab	4.8 ± 0.8 ab	5.8 ± 0.4 ab	6.0 ± 1.0 ab	6.5 ± 0.4 ab	6.6 ± 0.7a
A_Hay	5.0 ± 0.6d	5.2 ± 1.1d	6.5 ± 0.7cd	7.2 ± 1.2bcd	8.8 ± 1.2bc	9.8 ± 0.9 ab	10.5 ± 0.8a
A_Meat Broth	11.4 ± 1.3a	10.5 ± 0.7 ab	7.5 ± 1.0bc	5.9 ± 1.2cd	3.9 ± 1.4d	3.8 ± 0.4d	2.8 ± 0.4d
BT_Sweet	7.7 ± 0.3a	7.3 ± 0.6 ab	6.6 ± 0.8abc	5.2 ± 0.8cd	5.7 ± 1.1bcd	4.7 ± 1.1d	5.2 ± 0.5cd
BT_Bitter	5.4 ± 0.4a	5.4 ± 0.8a	5.4 ± 0.5a	5.6 ± 0.3a	5.8 ± 0.4a	6.2 ± 0.9a	6.5 ± 0.5a
BT_Umami	9.9 ± 0.8a	9.9 ± 0.9a	7.7 ± 1.1 ab	6.7 ± 1.0b	6.7 ± 0.3b	5.5 ± 0.6b	5.8 ± 0.6b
F_Watery	4.1 ± 0.2bc	4.0 ± 0.4c	5.9 ± 1.0abc	7.5 ± 0.3a	7.4 ± 0.7a	7.4 ± 0.9a	6.9 ± 0.8 ab
F_Corn	6.4 ± 0.5a	6.3 ± 0.5a	4.9 ± 0.9a	4.1 ± 0.6a	4.2 ± 0.4a	4.3 ± 0.8a	4.5 ± 0.6a
F_White Asparagus	6.9 ± 0.8a	6.2 ± 0.7 ab	5.6 ± 1.1 ab	5.0 ± 1.0 ab	5.4 ± 0.6 ab	4.5 ± 0.9b	4.6 ± 0.4b
F_Hay	6.0 ± 0.8cd	5.3 ± 1.0d	7.0 ± 1.4bcd	7.9 ± 0.1abcd	8.4 ± 0.8abc	9.1 ± 0.6 ab	10.2 ± 0.2a
F_Oxidised	2.3 ± 0.7a	2.5 ± 0.7a	2.4 ± 1.4a	2.7 ± 0.5a	3.0 ± 0.5a	5.0 ± 1.2a	4.8 ± 0.4a
F_Boiled Potato	7.4 ± 0.9a	7.7 ± 0.7a	8.5 ± 0.6a	9.1 ± 0.8a	9.1 ± 0.5a	8.7 ± 0.2a	8.6 ± 0.5a
F_Meat Broth	9.4 ± 0.3a	8.9 ± 0.7 ab	6.2 ± 0.4bc	4.3 ± 1.1cd	3.8 ± 0.6cd	3.2 ± 0.7d	3.1 ± 0.4d

*Panel averaged scores on the 15 cm intensity scale.

**Fig. 1:**
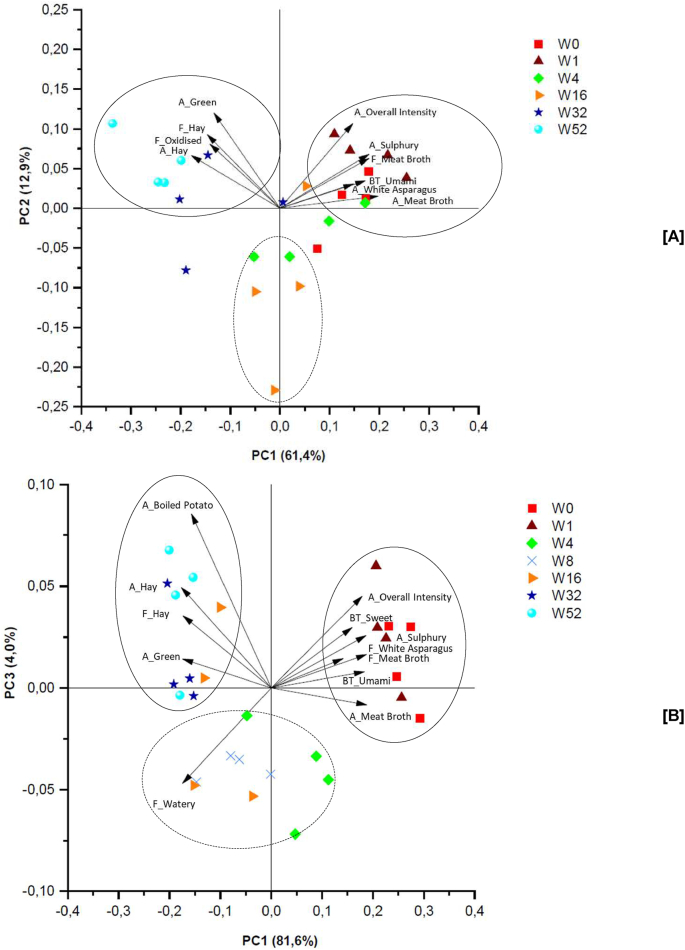
Principal component analysis (PCA) biplot of most important principle components (PC) for the significant sensory attributes and chickpea samples stored in aluminium pouches [A] and plastic pouches [B] at 20 °C for defined storage times between 0 and 52 weeks. W in legend stands for the weeks of storage. A = orthonasal aroma; BT = basic taste; F = flavour.

PCA was separately performed on chickpeas stored in aluminium and plastic pouches, including the sensory attributes changing significantly in the respective sample sets. PCA models with five and seven principal components were obtained, explaining 93.5% and 97.3% of the variance for the chickpeas in aluminium and plastic pouches, respectively. Visual representations of the PCA models using the most significant PCs are shown in Fig. 1. In these biplots, the samples stored for different times are represented with coloured objects, and the vectors represent the correlation loadings for the different sensory attributes. For both packaging materials, the first PC mainly explains the storage time from short to long storage from right to left. The other PC explains (to a limited extent) the ‘intensity’ of flavours and aromas in the samples, separating samples with intense aromas and flavours (e.g. sulphury, hay, etc.) from the samples that score lower in these attributes. For the plastic pouches, the samples with lower intensity were also characterised with higher scores for watery flavour, which was an indication for ‘tasteless’.

From the PCA biplots it appeared that several sensory attributes were correlated. The attributes for sulphury aroma, meat broth flavour and white asparagus flavour (in plastic pouches only) appeared to be highly correlated. This is not completely surprising as in literature sulphur compounds sometimes are described as meaty or asparagus-like. For example dimethyl sulphide has been described as having a cooked, asparagus-like, cabbage like, sulphury odour, while dimethyl trisulphide is described as meaty, cabbage-like and sulphury (Belitz et al., 2004; Kaczmarska et al., 2018). In the chickpeas stored in plastic pouches, the overall intensity was negatively correlated to watery flavour. Since watery flavour was defined as the flavour of plain cooked bulgur, it represents the lack of significant flavours. Therefore, it is not surprisingly that this attribute was negatively correlated to overall intensity. For the attributes hay and meat broth, both aroma and flavour were assessed. Although some difference in changes between the samples were found, still the attributes lay in the same group of samples. Therefore, hay and meat broth aroma attributes appeared to be good indicators for the corresponding flavours and vice versa.

Looking at the position of the samples in the PCA plots, a clear trend over storage time was observed. For both packaging materials two distinct sample groups were identified based on the sensory attributes, indicated by the full circles in the biplots, and one intermediate sample group was identified, indicated with the dotted circle in the biplots. These three groups represent ‘fresh samples’ (0–4 weeks stored), ‘intermediate stored samples’ (4–16 weeks stored) and ‘long stored samples’ (16–52 weeks).

The fresh samples from both packaging materials were characterised with an intense, sulphury and meat broth aroma, a meat broth flavour and an umami taste, compared to the longer stored samples. The samples from aluminium pouches were additionally characterised by white asparagus aroma, the samples from plastic pouches by white asparagus flavour and sweet taste. During sterilisation, Maillard related reactions could take place, which could give rise to sulphur containing compounds (Van Boekel, 2006). These type of compounds could possess meaty, sulphury, asparagus type of aromas (Goniak and Noble, 1987; Van Boekel, 2006). Strecker degradation reactions during sterilisation can result in sulphur-containing heterocyclic compounds, which contribute to strong cooked asparagus aromas. Additionally, decarboxylation of asparagusic acid could have resulted in the formation of the unstable 1,2-dithiacyclopentene, which possess a cooked asparagus smell (Tressl et al., 1977). This could explain why the asparagus intensity is high after sterilisation but decreases with longer storage (see below).

In contrast, the intermediate samples had lower intensities for most attributes and were (in plastic pouches) more closely correlated to watery flavour (lack of distinct flavours). This indicates that the components causing the sulphury, meat broth notes in the fresh samples have been degraded during intermediate storage time (4–16 weeks), which might be the result of oxidative rearrangements and degradation during storage (Kebede et al., 2015). More targeted studies on these sulphury notes are needed to understand the changes from sulphury flavours to watery flavour during storage.

The third group, representing long-stored samples was characterised by green and hay aroma and hay flavour. It seems that the formation of components inducing these attributes increased with storage. Alcohols and aldehydes between C_6_–C_9_, which can be formed during lipid degradation, are often associated with green notes (Fauconnier et al., 1999).

In the samples from aluminium pouches an ‘oxidised’ off-flavour additionally increased after longer storage, in the samples from plastic pouches boiled potato aroma increased. It evidently was expected that oxidised (off-)flavours would be reduced when storage took place with reduced oxygen permeability (i.e. in aluminium pouches). It is possible that the flavour that the panel described as ‘oxidised’ was actually another (off-)flavour. Since the sensory reference for oxidised flavour attributes was rustic potato chips of which the bag was opened overnight, there might be a correlation between the oxidised attribute and the boiled potato aroma attribute.

##### 3.1.3.2 Influence of packaging material on the sensory attributes of sterilised chickpeas

As previously mentioned, the samples in both packaging materials showed similar sensory trends during 52 weeks storage. However, some differences were observed. In the chickpeas contained in aluminium pouches, 10 out of 17 attributes changed significantly during storage, for the samples in plastic pouches this were 12 attributes. It appeared that slightly less and different changes occurred during storage at lower oxygen availability.

All samples of both packaging materials were compared together, to evaluate if sensory changes took place significantly faster in one of the two packages. Based on the biplots in Fig. 1, it was suggested that volatile changes occurred slightly faster at higher oxygen availability. Although the samples in the two packaging materials were characterised by similar attributes, the breakdown of sulphur related compounds seemed faster, as the samples stored for 4 weeks in plastic pouches already moved further away from the vectors representing these attributes. Moreover, the formation of hay and green related compounds appeared to be slightly faster in the plastic pouches. Here, the 32–52 weeks stored chickpeas were clearly located close to the vectors representing the hay and green related attributes and even the chickpeas stored for 16 weeks were partly located in this group. In the samples stored in aluminium pouches, the vectors representing the green and hay related attributes were only directed towards the 52-week stored samples and part of the 32-week stored samples.

**Fig. 2:**
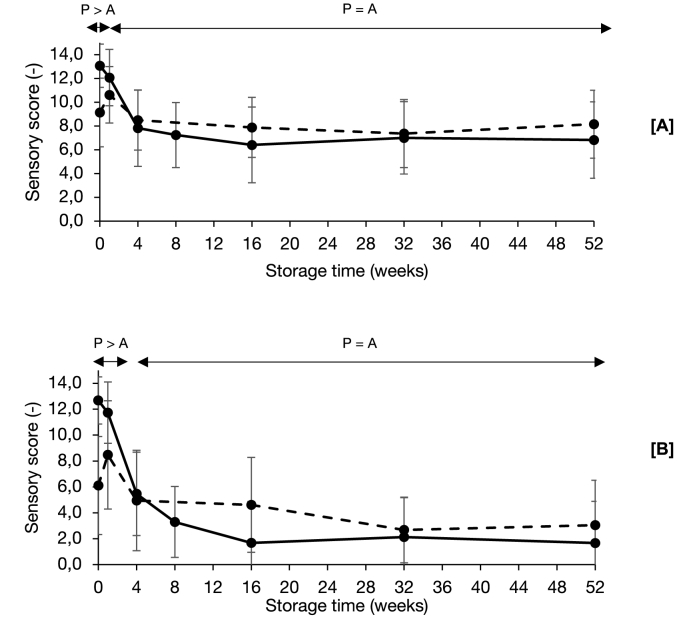
Plotted intensity scores for overall intensity [A] and sulphury [B] aroma of chickpeas stored in aluminium (dashed lines) and plastic (full lines) pouches up to 52 weeks. Standard deviations of repetitions are given. Significant differences (α = 0.05) are indicated with arrows, where ‘P’ stands for the plastic pouches and ‘A’ for the aluminium pouches.

Nonetheless, based on the data analysis on the combined data set, only for two attributes, the overall intensity and sulphury aroma, significant differences were found between samples with the same storage time in the two different packaging materials (e.g. chickpea from aluminium pouches at 1 week storage versus chickpea from plastic pouches at 1 week storage). The sensory scores of the overall intensity and sulphury aroma for both oxygen availabilities are plotted in Fig. 2. Remarkably, these significant differences were only visible at the beginning of storage. For the overall intensity aroma, only the chickpeas stored for 0 weeks, scored significantly higher in the plastic pouches, compared to the aluminium pouches. For the sulphury aroma, the chickpeas in the plastic pouches scored higher at 0 and 1 weeks of storage. This indicated that differences were induced by differences in oxygen availability during processing, rather than during storage. Possibly, during sterilisation at higher oxygen availability, more aldehydes are formed by thermal lipid oxidation, which could participate in Maillard-type reactions, to form sulphur containing volatile compounds such as thiophenes and thiazoles, which could contribute to intense, sulphury aromas (Kerth and Miller, 2015).

During the 52-week storage, sulphur compounds degraded in chickpea samples from both packaging materials. This breakdown did not seem to be influenced by the oxygen availability, as similar values were reached during storage. Therefore, it was concluded that oxygen availability does not largely influence the volatile profile of sterilised chickpeas stored up to 52 weeks at room temperature protected from light.

### 3.2 Gas chromatography – mass spectrometry - olfactometry analysis

The chickpea samples at the beginning (week 0) and end (week 52) of storage in both packaging materials were analysed using GC-MS-O. Although over 100 different volatile compounds have been reported in sterilised chickpeas (Noordraven et al., 2021), it is not yet known which of these volatiles are odour-active and thus contribute to the aroma of the sterilised chickpeas. In this study, 40 odour-active compounds were found among the four samples. These odour-active volatiles, their described odours, retention indices, NIF, IS, OI, and relative peak areas are shown in Table 4. The identified compounds mainly consisted of aldehydes (33%), nitrogen containing compounds (20%) and ketones (13%). The chickpeas stored in aluminium pouches possessed slightly less odour-active compounds compared to those stored in the plastic pouches. In the fresh chickpeas, 37 and 40 odour-active volatiles were and in the 52-week stored chickpeas 35 and 38 odour-active volatiles were detected, for chickpeas stored in aluminium and plastic pouches, respectively.

**Table 4:** Odour descriptions, compound identifications, retention indeces, nasal impact frequencies, intensity scores, odour importance scores and relative peak areas of the discrete aroma-active compounds detected with GC-MS-O in sterilised chickpeas stored for 0 and 52 weeks in aluminium (A0 and A52, respectively) and plastic pouches (P0 and P52, respectively). ND = not detected.

	Odour description	Compound	Identif-ication^c^	RI_GC_^d^	RI_snif_^e^	Nasal impact frequency (%)				Intensity Score (%)				Odour importance score (%)				Relative peak area^f^			
						A0	A52	P0	P52	A0	A52	P0	P52	A0	A52	P0	P52	A0	A52	P0	P52
1	Fruity, solvent	2-Butanone	m, r, a, s	908	919	89	100	0	56	55	42	0	23	70	65	0	36	0.12 ± 0.02	0.11 ± 0.02	0.14 ± 0.04	0.13 ± 0.05
2	Chemical, solvent	2-Methyl-2-propanol	m, r, a	916	917	11	0	56	44	8	0	24	20	9	0	36	30	0.16 ± 0.03	0.19 ± 0.07	0.16 ± 0.06	0.18 ± 0.05
3	Caramel	Diacetyl	m, r, a, s	975	981	78	78	78	78	48	50	48	53	61	63	61	64	0.01 ± 0.02	0.004 ± 0.002	0.01 ± 0.01	0.002 ± 0.002
4	Roasted	Unidentified	–	1053	1051	44	67	56	33	25	23	15	10	33	39	29	18	0.05 ± 0.01	0.03 ± 0.01	0.04 ± 0.02	0.08 ± 0.06
5	Fruity, pineapple	Ethyl butyrate^a^	r, a, s	ND	1068	78	44	56	78	46	19	32	38	60	29	42	54	ND	ND	ND	ND
6	Caramel	2,3-Pentanedione^a^	r, a	ND	1075	56	33	44	33	29	9	30	13	40	18	37	21	ND	ND	ND	ND
7	Grass, green, flower	Hexanal	m, r, a, s	1096	1088	100	89	89	89	61	46	55	52	78	64	70	68	1.77 ± 0.32	0.29 ± 0.08	1.14 ± 0.25	0.25 ± 0.16
8	Plastic, rubber	2-Methyl-1-propanol	m, r, a, s	1110	1110	67	67	56	67	42	37	35	40	53	49	44	51	0.16 ± 0.03	0.07 ± 0.01	0.18 ± 0.04	0.07 ± 0.01
9	Plant, grass	2-Butylfuran	m, r, a	1147	1147	0	33	44	22	0	16	21	10	0	23	30	15	0.004 ± 0.001	0.004 ± 0.004	0.01 ± 0.00	0.02 ± 0.01
10	Potato, plastic, beany	Unidentified	–	1172	1176	56	0	56	44	22	0	18	15	35	0	32	26	0.02 ± 0.00	0.01 ± 0.00	0.02 ± 0.00	0.03 ± 0.01
11	Vegetable, plants, sharp, rubber	Pyridine	m, r, a, s	1199	1205	56	67	56	56	26	30	28	21	38	45	39	34	0.41 ± 0.07	0.75 ± 0.11	0.95 ± 0.27	0.62 ± 0.21
12	Flower, perfume, paint	2-Hexenal	m, r, a	1217	1217	56	11	33	33	35	4	22	17	44	7	27	24	0.06 ± 0.01	0.03 ± 0.01	0.07 ± 0.01	0.04 ± 0.01
13	Off-flavour, cheesy^g^, socks^g^	3-Methyl-1-butanol	m, r, a, s	1227	1225	100	100	44	100	64	63	34	56	80	79	39	75	0.93 ± 0.06	0.25 ± 0.05	0.76 ± 0.10	0.79 ± 0.09
14	Minty^g^, candy, fruity	2-Pentyl-furan	m, r, a, s	1245	1247	11	44	56	22	8	18	22	13	9	28	35	17	0.08 ± 0.02	0.08 ± 0.01	0.40 ± 0.12	0.14 ± 0.04
15	Plant, beany, spicy	Unidentified	–	1254	1259	78	33	56	56	46	23	32	32	60	28	42	42	0.004 ± 0.004	0.01 ± 0.01	0.01 ± 0.01	0.002 ± 0.001
16	Citrus, orange	Octanal	m, r, a, s	1313	1313	89	56	89	78	57	38	55	44	71	46	70	58	0.05 ± 0.01	0.03 ± 0.01	0.09 ± 0.02	0.04 ± 0.02
17	Mushroom, vegetable	1-Octen-3-one	m, r, a, s	1324	1326	78	89	78	89	53	43	48	68	64	62	61	78	0.004 ± 0.001	0.002 ± 0.001	0.004 ± 0.002	0.005 ± 0.001
18	Broth^g^, cheesy^g^	(E)-2-Heptenal^b^	m, r, a, s	1349	1354	67	22	33	22	45	11	21	7	55	16	26	12	0.05 ± 0.02	0.02 ± 0.00	0.03 ± 0.01	0.03 ± 0.01
19	Popcorn, cooked rice	2-Acetyl-1-pyrroline	m, r, a	1362	1368	89	56	89	67	71	40	65	42	79	47	76	53	0.02 ± 0.00	0.02 ± 0.00	0.03 ± 0.00	0.02 ± 0.00
20	Popcorn, plant, vegetable	Unidentified	–	ND	1384	33	44	44	22	19	19	23	9	25	29	32	14	ND	ND	ND	ND
21	Plant, chemical, beany, off	Unidentified	–	1395	1401	89	78	22	78	39	45	15	39	59	59	18	55	0.003 ± 0.005	0.001 ± 0.001	0.001 ± 0.001	0.003 ± 0.002
22	Grassy, green, fruity, pungent	Nonanal	m, r, a, s	1417	1415	100	100	100	89	64	65	62	46	80	80	79	64	0.08 ± 0.03	0.06 ± 0.02	0.13 ± 0.04	0.06 ± 0.03
23	Minty, mushroom, grassy, fruity, flowery	Unidentified	–	1437	1437	56	44	67	44	34	19	41	15	44	29	52	26	0.01 ± 0.00	0.002 ± 0.002	0.04 ± 0.01	0.003 ± 0.003
24	Rubber, mint, methoxypyrazine	Unidentified	–	1445	1448	22	67	11	56	16	43	10	33	19	53	11	43	0.001 ± 0.001	0.002 ± 0.001	0.002 ± 0.002	0.003 ± 0.002
25	Beany, green, nutty, earthy	Acetic acid	m, r, a, s	1459	1460	89	78	100	89	75	62	74	70	82	70	86	79	0.01 ± 0.00	0.003 ± 0.005	0.01 ± 0.01	0.005 ± 0.002
26	Potato, beany, pea	Methional	m, r, a	1478	1480	67	89	33	67	38	51	20	37	50	67	26	50	0.01 ± 0.00	0.01 ± 0.00	0.01 ± 0.00	0.01 ± 0.00
27	Fries, potato, cheesy	Furfural	m, r, a	1483	1487	67	0	22	44	45	0	14	32	55	0	18	38	0.18 ± 0.03	0.18 ± 0.03	0.23 ± 0.07	0.26 ± 0.15
28	Plants, flower, vegetables	Decanal	m, r, a	1526	1528	67	67	33	22	44	38	21	11	54	50	26	15	0.03 ± 0.01	0.03 ± 0.01	0.04 ± 0.02	0.03 ± 0.02
29	Plants, nutty, pyrazine, rubbery, soap	Pyrrole	m, r, a, s	1532	1537	44	22	44	56	18	11	15	28	28	15	26	39	0.02 ± 0.02	0.01 ± 0.00	0.01 ± 0.00	0.01 ± 0.00
30	Flower, plant, pea	Benzaldehyde	m, r, a, s	1561	1559	67	67	89	100	45	46	55	60	55	55	70	77	0.30 ± 0.02	0.12 ± 0.02	0.13 ± 0.05	0.34 ± 0.03
31	Leather, lipstick, perfume	(E)-2-Nonenal	m, r, a, s	1562	1570	67	33	22	44	42	22	15	29	53	27	19	36	0.04 ± 0.03	0.03 ± 0.03	0.04 ± 0.03	0.09 ± 0.09
32	Pickled cucumber	(E)-2,(Z)-6-Nonadienal^a^	r, a	ND	1623	78	67	33	0	36	28	17	0	53	43	24	0	ND	ND	ND	ND
33	Mushroom, tofu, mould, herbs	Unidentified	–	ND	1631	0	0	56	56	0	0	32	31	0	0	42	41	ND	ND	ND	ND
34	Off, rubber	Benzonitrile	m, r, a, s	1649	1658	78	0	22	0	54	0	9	0	65	0	14	0	0.01 ± 0.00	0.002 ± 0.003	0.01 ± 0.00	0.01 ± 0.00
35	Flower, sweet, off-flavour	Butyrolactone^a^	r, a, s	ND	1665	44	44	67	56	19	26	42	33	29	34	53	43	ND	ND	ND	ND
36	Popcorn, cooked rice	2-Acetylthiazole	m, r, a, s	1689	1688	78	89	89	89	61	68	72	62	69	78	80	74	0.06 ± 0.04	0.06 ± 0.04	0.10 ± 0.06	0.11 ± 0.08
37	Beany, vegetables	Ethyl benzoate	m, r, a, s	1710	1707	67	33	56	33	45	17	29	10	55	24	40	18	0.02 ± 0.01	0.02 ± 0.01	0.02 ± 0.02	0.03 ± 0.01
38	Flower, plant, grain	Unidentified	–	ND	1716	0	67	33	0	0	35	15	0	0	48	22	0	ND	ND	ND	ND
39	Beans, oats, fish feed, roasted	Unidentified	–	ND	1719	0	0	44	56	0	0	29	27	0	0	36	39	ND	ND	ND	ND
40	Sweet, candy fruit	Damascenone^a^	r, a, s	ND	1881	78	33	22	78	45	16	13	40	59	23	17	56	ND	ND	ND	ND

a Tentatively identified.

b Possibly another compound is co-eluting making the aroma more broth-like.

c Identification: m: mass spectrum of compound in agreement with the NIST library; r: retention index in agreement with reported values in literature; a: aroma in agreement with reported aromas in literature; s: mass spectrum, retention index and odour in agreement with analytical standard of the identified compound.

d Retention index of the compounds derived on the DB-WAX column.

e Retention index of the compounds derived based on the moment the panellists noticed the compounds.

f Peak area of compounds relative to the peak area of the internal standard in Total Ion Chromatogram.

g This is a more uncommon descriptor for the aroma of this specific compound, but was confirmed by the panel by sniffing an analytical standard that underwent the same GC-O profile. Possibly, this more uncommon description was given by the panel, due to the limited training on vocabulary use, making descriptions less conform with the international flavour terminology.

The most important aroma-contributing compounds were selected based on the highest OI (>65%) in the different samples (Table 5). These compounds are expected to play an important role in the aroma of sterilised chickpeas, as they were frequently detected and were observed to have relatively intense aromas. The compounds comprise different aroma descriptors including grassy, green, popcorn and rice-like, fruity, citrus-like, earthy, potato-like, and beany off-odour. Some of these descriptors, such as green, potato, popcorn and earthy, were also mentioned in the sensory description (section 3.1.1**)**. All compounds were compared to literature and, when available, analytical standards, to ensure that the odour descriptions matched the identification of the compounds.

**Table 5:** Important odour-active volatile compounds isolated from the headspace of sterilised chickpeas stored for 0 and 52 weeks in aluminium (A0 and A52, respectively) and plastic (P0 and P52, respectively) pouches. Volatiles are ranked according to their average odour importance score.

	Compound	Odour description	Odour importance score (%)			
			A0	A52	P0	P52
1	Acetic acid	Beany, green, nutty, earthy	82	70	86	79
2	Nonanal	Grassy, green, fruity, pungent	80	80	79	64
3	2-Acetylthiazole	Popcorn, cooked rice	69	78	80	74
4	Hexanal	Grass, green, flower	78	64	70	68
5	3-Methyl-1-butanol	Cheesy, socks, off-flavour	80	79	39	75
6	1-Octen-3-one	Mushroom, vegetable	64	62	61	78
7	Benzaldehyde	Flower, plant, pea	55	55	70	77
8	2-Acetyl-1-pyrroline	Popcorn, cooked rice	79	47	76	53
9	Octanal	Citrus, orange	71	46	70	58
10	Methional	Potato, beany, pea	50	67	26	50

Some volatiles were only observed in the chickpea from one of the two packaging materials **(**Table 4**)**. Two unidentified compounds, compound 33 (mushroom, tofu, mould, herbs-like aroma) and compound 39 (beans, oats, fish feed, roasted aroma) were only observed in the chickpeas contained in plastic pouches. Since these type of aromas were not described to be present in significantly higher amounts in the chickpeas stored in plastic pouches (section 3.1), it is suggested that these compounds either did not significantly influence the aroma of the whole chickpeas packed in plastic pouches or interacted with other aroma compounds in the intact chickpeas, giving rise to a different aroma (Bott and Chambers, 2006).

For some volatiles, changes in OI took place over time. For several volatiles, such as 2-3-pentadione, octanal, (E)-2-heptenal, 2-acetylpyrroline, benzonitrile and ethyl benzoate, the OI decreased during storage for chickpeas from both packaging materials (Table 4). This was in agreement with a decrease in peak area for most of these compounds, although the peak area of ethyl benzoate in the aluminium pouches increased over time. The aromas of these compounds were described using various descriptors including caramel, citrus, mushroom, broth, pickled cucumber, beany and off-aroma. The OI for compound 24 and methional increased during storage. Compound 24 was described as having a rubbery and minty aroma, which was not specifically described in the descriptive sensory analysis. Methional, was described as having a potato-like aroma. This aroma was also increasing during the descriptive sensory analysis in the plastic pouches. However, the peak areas for methional appeared to be similar at the beginning and end of the storage period, instead of increasing.

Remarkably, the OI of the volatile compounds in Table 5 are generally similar for all the four samples. This indicates that the most important volatiles for chickpea aroma are not largely influenced by storage time and packaging material. Hence, it seems that most of the volatile compounds presented in Table 5 cannot be directly linked to the sensory results, where the changes over storage time were found to be more distinct. However, some small changes in OI were observed. As mentioned before, the OI of methional was increasing over time, especially for the chickpeas stored in plastic pouches. This was in line with the sensory test where the aroma of boiled potato was increasing in intensity during storage time in chickpeas stored in plastic pouches. Methional can be formed by the degradation of methionine (Cheng et al., 2020). For other compounds, changes could not be linked to the sensory results in section 3.1. For example, the intensity of the ‘green components’, hexanal, nonanal and acetic acid show a clear reduction in peak area and OI based on the GC-MS-O analysis, while in the descriptive analysis an increased green aroma was observed. Although acetic acid in literature is mainly described to have sour, vinegar-like aromas, GC-MS-O analysis of the analytical standard showed that it additionally gave a green, pea-like aroma. Two other compounds, 2-acetyl-pyrroline and octanal, which give popcorn-like and citrus-like aromas, respectively, additionally decreased during storage based on their OI and peak areas. These aroma types were not mentioned to be changing in the descriptive analysis. 2-Acetylpyrroline can be formed from the amino acids proline and ornithine during processing (Belitz et al., 2004; Bredie et al., 1996). During storage, the compound might have oxidised forming compounds like 2-acetylpyrrole, although this was not detected during with GC-MS-O (Belitz et al., 2004). Octanal can be formed during lipid oxidation during the sterilisation process and can react further during storage to for example octanoic acid (Corberán et al., 2014; Whitfield and Mottram, 1992).

In general, not many clear links could be made between the results of the sensory analysis and specific odour-active compounds analysed using GC-MS-O. This can have several reasons. Most likely, not all volatile compounds present in the chickpeas have been captured using the dynamic head-space extraction. For examples, highly volatile sulphur compounds might have been lost, which possibly could have explained the changes in intense and sulphury aromas in the intact chickpeas. Furthermore, the odour characteristics of volatile compounds tend to be concentration dependent. This means that if the concentration of a specific compound increases or decreases, different aroma descriptors could be observed (Vara-Ubol et al., 2003). During the dynamic headspace extraction, volatile compounds are concentrated on a trap. Consequently, the concentrations of volatiles observed in the GC-MS-O analysis might not be comparable to the concentrations perceived in intact chickpeas. Possibly the detected odour-active compounds differently contributed to the aroma in the intact chickpeas and therefore no clear link between GC-MS-O and descriptive analysis was found. Additionally, a mixture of two or more volatiles at lower concentrations can potentially give stronger perceived aromas than isolated compounds at higher concentrations (Bott and Chambers, 2006). In the GC-MS-O, all volatiles were presented as isolated compounds, in contrast to the intact chickpeas where all volatiles were perceived simultaneously. Therefore, it is possible that a combination of for example several ‘green’ related volatiles causes the chickpeas to have a green aroma which increased over storage, while the individual compounds in the GC-MS-O gave an opposite indication. Lastly, the intact chickpea matrix might obstructed the release of volatile compounds during the descriptive analysis, in contrast to the GC-MS-O analysis where chickpeas were pureed prior to volatile extraction (Aguilera, 2019). Due to all the above-mentioned reasons, the sensory profile of intact sterilised chickpeas cannot be linked to and explained by changes in individual aroma-active components.

## 4 Conclusions

For the first time, differences in the aroma and flavour of sterilised chickpeas stored up to 52 weeks at different oxygen availability were analysed using sensory descriptive analysis and GC-MS-O. The sensory attributes that described the overall aroma and flavour of sterilised chickpeas included sulphury, meaty, green, hay and potato-like. During 52 weeks storage, significant sensory changes were induced in the chickpeas. Intense, sulphury, meat broth-like characteristics decreased, while green, hay-like characteristics increased during storage. Additionally, some potato-like flavours and ‘oxidised’ off-flavours were formed during longer storage. Except for the first week of storage, minimal differences were observed between the chickpeas stored in the two packaging materials, indicating that the permeability of the packaging did not noticeably impact the sensory attributes of the sterilised and stored chickpeas. Therefore, it was concluded that the most sustainable or economically favourable packaging material can be used.

A total of 40 odour-active volatiles were found in the sterilised chickpea samples. The 10 compounds that were considered to have the highest impact on the chickpea aroma, were determined based on the detection frequency and intensity. These compounds included aldehydes, sulphur and nitrogen containing compounds as well as a ketone, alcohol and acid, giving rise to different aromas such as grassy, vegetable-like, beany, popcorn-like, earthy, potato-like and fruity. Changes in sensory properties could not be directly linked to individual volatile compounds, potentially due to the fact that not all volatile compounds could be captured using the dynamic headspace approach, interaction effects, matrix effects or differences in volatile concentrations for the isolated compounds.

This study provides useful insight into the aroma and flavour characteristics of sterilised chickpeas, stored up to 52 weeks. This information forms the scientific basis for future research into chickpea flavour, potentially leading to more acceptable chickpeas and therewith to an increased global legume intake. Targeted volatile analysis is recommended to be able to relate the descriptive sensory results to specific volatile compounds. Additional sensory consumer studies are recommended to investigate whether the differences found in sensory attributes during 52-week storage also affect the overall acceptability of the chickpeas and whether a specific flavour is most desired.

## Funding sources

This research project is part of the FOODENGINE project and has received funding from the 10.13039/100010661European Union’s Horizon 2020 Research & Innovation Programme under the Marie Skłodowska-Curie Grant Agreement No. 765415.

## Data linking

The data used for the graphs in this paper are available on https://doi.org/10.5281/zenodo.5596737.

## CRediT authorship contribution statement

**Laura E.C. Noordraven:** Conceptualization, data collation, Formal analysis, data interpretation, Writing – original draft. **Mikael A. Petersen:** Conceptualization, data interpretation, Writing – review & editing. **Ann M. Van Loey:** Conceptualization, data interpretation, Writing – review & editing, Supervision. **Wender L.P. Bredie:** Conceptualization, Formal analysis, data interpretation, Writing – review & editing, Supervision.

## Declaration of competing interest

The authors declare that they have no known competing financial interests or personal relationships that could have appeared to influence the work reported in this paper.

## Abbreviations

GC-MS-O: Gas Chromatography – Mass Spectrometry – Olfactometry
IS: Intensity score
NIF: Nasal impact frequency
OI: Odour importance score
PC: Principal component
PCA: Principal component analysis
RMSECV: Root mean square error of cross validation
